# Sensorimotor gating impairments induced by MK-801 treatment may be reduced by tolerance effect and by familiarization in monkeys

**DOI:** 10.3389/fphar.2015.00204

**Published:** 2015-09-22

**Authors:** Patricia G. Saletti, Rafael S. Maior, Etsuro Hori, Hisao Nishijo, Carlos Tomaz

**Affiliations:** ^1^Primate Center and Laboratory of Neurosciences and Behavior, Department of Physiological Sciences, Institute of Biology, University of Brasilia, Brasilia, Brazil; ^2^System Emotional Science, Graduate School of Medicine and Pharmaceutical Sciences, University of Toyama, Toyama, Japan; ^3^Neurosciences Research Group, Universidade CEUMA, São Luís, Brazil

**Keywords:** PPI, dizocilpine (MK-801), schizophrenia, NMDA receptor, habituation

## Abstract

Dizocilpine (MK-801) is a non-competitive NMDA antagonist that induces schizophreniclike effects. It is therefore widely used in experimental models of schizophrenia including prepulse inhibition (PPI) impairments in rodents. Nevertheless, MK-801 has never been tested in monkeys on a PPI paradigm. In order to evaluate MK-801 effects on monkeys’ PPI, we tested eight capuchin monkeys (*Sapajus* spp.) using three different doses of MK-801 (0.01; 0.02; 0.03 mg/kg). Results show PPI impairment in acute administration of the highest dose (0.03 mg/kg). PPI impairment induced by MK-801 was reversed by re-exposure to the PPI test throughout treatment trials, in contrast with rodent studies. These results indicate that tolerance effect and familiarization with PPI test may reduce the sensorimotor gating deficits induced by MK-801 in monkeys, suggesting a drug-training interaction.

## Introduction

Prepulse inhibition (PPI) impairment is characteristic of schizophrenic patients (Braff et al., 2001a; Ludewig et al., 2003), and also usually observed in rodents after MK-801 injection (Long et al., 2006; Arai et al., 2008; Ishii et al., 2010; Gururajan et al., 2011; Gomes et al., 2014; Khella et al., 2014; Park et al., 2014). PPI test is characterized as a slight stimulus (prepulse) presented at a specific interval before the startling stimulus (pulse), leading to a reduction of the startle response. In this sense, PPI allows the evaluation of sensorimotor gating mechanisms through behavioral responses (Graham, 1975; Braff and Geyer, 1990; Geyer et al., 2001). Deficits in PPI reflect sensorimotor gating abnormalities that are prominent in schizophrenic patients (Braff et al., 2001a,b; Mackeprang et al., 2002; Kumari et al., 2003; Ludewig et al., 2003; Preuss et al., 2011). Since PPI test is a simple and direct way to measure a deficit in information processing, it is largely used in animal models, especially in rodents (e.g., Long et al., 2006; Arai et al., 2008; Ishii et al., 2010; Gururajan et al., 2011; Gomes et al., 2014; Khella et al., 2014; Park et al., 2014).

Hypofunction of glutamatergic receptors has been regarded as one of the explanations for the psychopathology of schizophrenia. As others NMDA antagonists—phencyclidine (PCP) and ketamine—MK-801 is extremely common in schizophrenia model studies. This is due to the fact that these drugs trigger schizophrenic-like effects as seen in several studies with rodents. It has been demonstrated that MK-801 induces recognition memory impairments, hyperactivity and hyperlocomotion (Bradford et al., 2010; Park et al., 2014; Basurto et al., 2015), learning, memory and spatial memory impairments (Hikichi et al., 2013; Karamihalev et al., 2014), social recognition deficits (Yoshimi et al., 2015), as well as impairments in cognitive set-shifting (Svoboda et al., 2015), and prepulse inhibition disruption (PPI) in rodents (Long et al., 2006; Arai et al., 2008; Ishii et al., 2010; Gururajan et al., 2011; Gomes et al., 2014; Khella et al., 2014; Park et al., 2014).

Studies with non-human primates have demonstrated that MK-801 is effective at inducing schizophrenic-like effects. Ogura and Aigner (1993), using rhesus monkeys (*Macaca mulatta*) demonstrated that MK-801 impairs visual recognition memory. In the same way, Buffalo et al. (1994) performed an Operant Test Battery, also in rhesus using MK-801. They showed MK-801 effectively disrupted animals accuracy in learning, motivation, short-term memory, and color discrimination tasks. Working memory is also impaired by acute and chronic MK-801 treatment in rhesus (Tsukada et al., 2005). Likewise, in common marmosets (*Callithrix jacchus jacchus*), MK-801 impaired acquisition of shape discrimination and visuospatial conditional tasks using the Wisconsin General Test Apparatus (Harder et al., 1998; Harder and Ridley, 2000). A more recent study with rhesus monkeys evidenced that a single administration of MK-801 at doses of 0.02 and 0.04 mg/kg was able to impair spatial working memory (Wang et al., 2012).

To our knowledge, the effects of MK-801 on PPI have not yet been investigated in primates. Since there are significant morphological and neuropharmacological differences between rodents and primates (e.g., Maior et al., 2011), we employed a whole-body PPI protocol (Saletti et al., 2014) in capuchin monkeys (*Sapajus* spp.) to evaluate MK-801 effects on sensorimotor gating.

## Materials and Methods

### Ethics Statement

This study was approved by the Animal Ethics Committee of the Institute of Biology, University of Brasilia (UnBDOC no 131791/2013). Furthermore, the procedures were conducted according to guidelines of the Brazilian Society of Animal Experimentation and followed the Principles of Laboratory Animal Care (NIH publication no. 85-23, revised 1996).

Experiments were performed in Primate Center at University of Brasilia, Brazil. Animals were allocated into pairs or triads in home cages (3 × 3 × 1.8 m) with natural substrate, rope swings and nest boxes. In their home cages, they were given access to food twice a day, once early in the morning and once at the end of the day; water was offered *ad libitum* by automatic drinking tap nozzle. The subjects were always under natural conditions of lightness and temperature. No animal was submitted to any kind of suffering. Beyond that, in order to minimize the stress of life in captivity, environmental enrichment for the animals is provided in the Primate Center. It is important to emphasize that no subject has been euthanized after this study.

### Subjects

Eight capuchin monkeys (*Sapajus* spp.) were employed in this study, 6 females and 2 males, weighting between 2.5 and 5 kg. Seven animals had been used in PPI test previously (Saletti et al., 2014), and only one female was naïve. Nonetheless, no animal was previously exposed to the experimental drugs. All experiments were conducted between 8 and 12 am, 5 days a week. No food or water deprivation has been enforced, except during trials.

### Startle Measurement

Prepulse inhibition tests were performed inside a primate chamber (60 × 30 × 30 cm), built in transparent acrylic material of 15 mm thick, placed above a wooden box (45 × 40 × 40 cm). Animals were placed inside the chamber with its head out through an adjustable neck hole. Two speakers (Foster Model FT96H Frequency band; 4 KHz∼30 KHz) were attached to a head box (30 × 30 × 25 cm) on the top of the chamber. The speakers were kept each at a distance of 10 cm of the monkey’s head and were connected to a sound generator (O’Hara & Co., Ltd., Tokyo). On the bottom of the chamber, an accelerometer was (Inntechno Japan Co.ltd., Model: BDK3) connected to an amplifier (O’Hara & Co., Ltd.) which captured animal’s whole-body movement. The whole system was connected to a recording software (Animal Startle—PCI 6024E, developed by O’Hara & Co., Ltd.), interfaced with Windows XP operational system (for more details see, Saletti et al., 2014).

Prepulse inhibition tests were conducted in an acoustic isolated room next to the subjects’ home cage. Inside the test room, a permanent white noise was generated (65 dB) and a video camera (Model Clone #1004124) was used to monitor the animal during tests for animals’ safety.

### Drug

MK-801 (0; 0,01; 0,02; 0,03 mg/kg—Sigma-Aldrich, Brazil) was dissolved in Tween 80 (Sigma-Aldrich, Brazil) and 0.9% saline (1:19) administered intramuscularly (i.m.) in a volume of 1 ml/kg. All doses were based in studies with non-human primates (Buffalo et al., 1994; Harder et al., 1998).

### Procedures

Each session test consisted in 10 equal and consecutive blocks of 3 pseudorandomized stimuli each (pulse-alone, 115 dB, 40 ms duration; prepulse-alone, 80 dB, 20 ms duration and pulse-prepulse, 120 ms interval). Startle response was recorded as the maximum peak amplitude over 600 ms after each presentation.

Prepulse inhibition sessions were carried out with a 2-week interval. A week in which the subject received either vehicle (VEH) or any dose of MK-801 was considered a test-week. Therefore, the washout period for MK-801 was, at least 2 weeks. Subjects were divided into two groups: G1 received vehicle (VEH) at the first test-week and G2 received VEH at the fourth test-week. In the remaining test-weeks (G1: second–fourth test-weeks; G2: first–third test-weeks), they received three different doses of MK-801 (0.01, 0.02, 0.03 mg/kg i.m.) randomly assigned once a test-week. Each group comprised four subjects. PPI test was performed 20 min after MK-801 administration.

### Statistical Analysis

All statistic tests were conduct in IBM SPSS Statistics Version 20.

Shapiro Wilk Test was performed to verify the normality of the data. Since the data did not show normal distribution, Friedman’s Test was performed to examine the effects of MK-801 on startle amplitude and percentage of PPI.

Wilcoxon Rank Sum Test was performed to analyze independent data of percentage of PPI in temporal effect of MK-801, and Wilcoxon Signed Rank Test to examine dependent data in temporal effect of the drug.

To normalize the data, we calculated the percentage of inhibition of the startle response for each subject by the following formula: 100 × (pulse-alone – prepulse-pulse)/pulse-alone as done in previous PPI studies (Winslow et al., 2002, 2007; Saletti et al., 2014).

Results with *p* ≤ 0.05 were considered statistically significant. All data are represented as the mean of startle amplitude or percent of PPI ± standard error of mean ( ± SEM).

## Results

**FIGURE 1:**
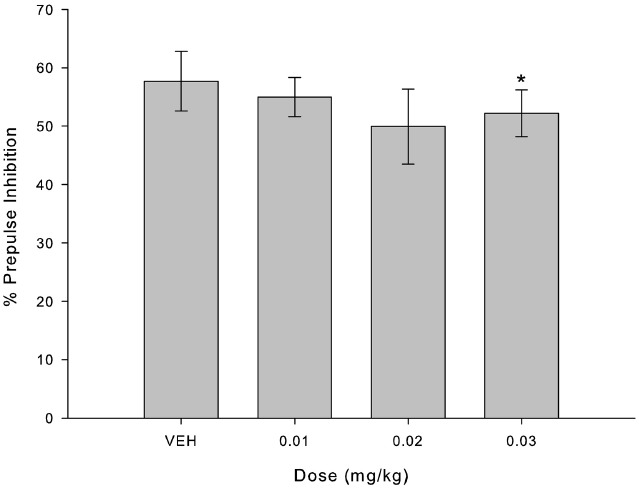
**MK-801 disrupted percent of PPI only in higher dose (0.03 mg/kg) compared to VEH.** Bars indicate percent of inhibition of startle response (mean ± SEM, *n* = 8) for each administrated dose. *Indicates significant difference between 0.03 mg/kg and VEH (*p* = 0.002) in Friedman’s test.

Figure 1 indicates the percentage of inhibition for each randomly assigned administration (VEH and three doses of MK-801), regardless of experimental group. Friedman’s Test indicated a decrease in percentage of inhibition in comparison with VEH only for the higher dose of MK-801 (0.03 mg/kg; X^2^ = 9.800; *p* = 0.002; Figure 1, *n* = 8).

**FIGURE 2:**
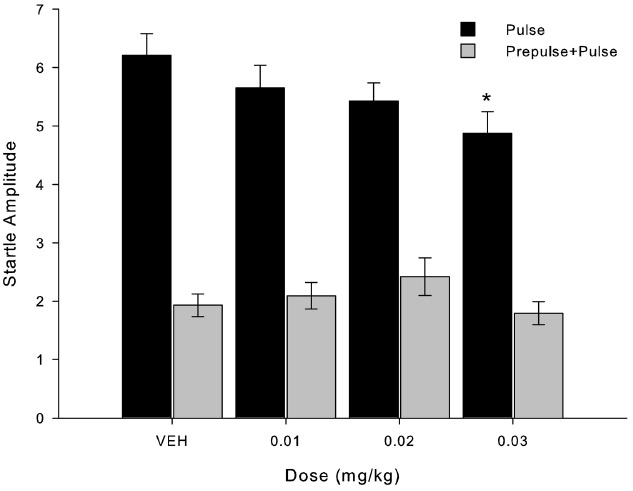
**MK-801 decreased startle response amplitude after administration of the higher dose (0.03 mg/kg) compared to VEH.** Bars indicate startle response amplitude (mean ± SEM, *n* = 8) when presented pulse-alone trials (black bars) and prepulse+pulse trials (gray bars). *Indicates significant difference in pulse-alone trials between 0.03 mg/kg and VEH (*p* = 0.025) and 0.01 mg/kg (*p* = 0.014) in Friedman’s test.

In Figure 2, we show the mean startle response amplitude when pulse-alone and prepulse+pulse are considered separately, again regardless of experimental group. In pulse-alone situation, startle response differs only from VEH at the dose of 0.03 mg/kg (X^2^ = 5.000; *p* = 0.025, *n* = 8). In prepulse+pulse stimuli, no dose was statistically different from VEH (X^2^ = 7.738; *p* = 0.052, *n* = 8).

**FIGURE 3:**
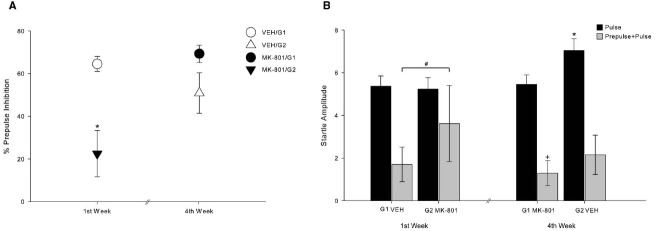
**Habituation effect of MK-801 over repeated PPI sessions. (A)** Circles indicate percent of PPI of G1 group (*n* = 4), and triangles, of G2 group (*n* = 4). White symbols indicate response after VEH administration and black symbols indicate response after MK-801 administration, regardless of dose (mean ± SEM). *Indicates significant difference of MK-801/G2 compared to all of other. (MK-801/G2 vs. VEH/G1, *p* < 0.001; MK-801/G2 vs. MK-801/G1, *p* < 0.001; MK-801/G2 vs. VEH/G2, *p* = 0.032). **(B)** At the first test-week, startle amplitude on pulse trials did not differ between groups, unlike on prepulse+pulse trials. Bars indicate startle response amplitude. Black bars demonstrate startle response on pulse-alone trials, and gray bars demonstrate startle response on prepulse-pulse trials. *Indicates significant difference on pulse-alone trials between VEH/G2 and all other (*p* < 0.036). +Indicates significant difference on prepulse-pulse trials between MK-801/G1 and all other (*p* < 0.032). #Indicates statistical difference on prepulse+pulse trials between VEH/G1 and MK-801/G2 (*p* = 0.042).

In our test procedure, we used a repeated treatment design in which all subjects underwent the same treatments. As explained above, we divided the animals in two groups (G1 and G2) and compared the PPI response between these two groups at the first and fourth test-weeks. This procedure was conducted in order to evaluate a possible temporal effect of MK-801 administration. Figure 3 shows the temporal effect of MK-801 administration. A deficit in the percent of PPI can be seen in animals that received MK-801 at the first test-week (MK-801/G2, *n* = 4), regardless of the dose that each monkey received, in comparison to animals that received VEH at the first test-week (VEH/G1, *n* = 4) (Z = –3.830; *p* < 0.001). Additionally, there was an increase in PPI after repeated injections of MK-801 and repeated PPI tests (Z = –4.359; *p* < 0.001), as observed in PPI of MK-801/G2 and MK-801/G1. A statistical difference was also observed between MK-801 at first test-week (MK-801/G2) and VEH at fourth test-week (VEH/G2) (Z = –2.151; *p* = 0.032). There was no statistical difference between either VEH administrations regarding percentage of inhibition (Z = –0.414; *p* = 0.684). Also, no difference was found between VEH at fourth test-week (VEH/G2) and MK-801 at fourth test-week (MK-801/G1) (Z = –0.799; *p* = 0.429), nor between VEH at first test-week (VEH/G1) and MK-801 at fourth test-week (MK-801/G1) (Z = –1.116; *p* = 0.265; Figure 3A).

Figure 3B shows the mean startle response amplitude when pulse-alone and prepulse+pulse are considered separately. In pulse-alone trials, startle response of VEH at fourth test-week (VEH/G2) was different from all other pulse-alone (vs. VEH/G1: Z = –2.223, *p* = 0.026; vs. MK-801/G2: Z = –2.850, *p* = 0.004; vs. MK-801/G1: Z = –2.098, *p* = 0.036). In prepulse+pulse trials, MK-801 at fourth test-week was statistically different from all others (vs. VEH/G1: Z = –2.139, *p* = 0.032; vs. VEH/G2: Z = –3.662, *p* < 0.001; vs. MK-801/G2: Z = –2.560, *p* = 0.010). Moreover, VEH at first test-week (VEH/G1) differs from MK-801 at first test-week (MK-801/G2) (Z = –2.035, *p* = 0.042).

## Discussion

The present study shows the effects of MK-801 on capuchin’s PPI response. Pretreatment with MK-801 has induced a decrease in PPI, as well as in startle amplitude at the highest dose (0.03 mg/kg). Other MK-801 studies in primates using different paradigms also indicate that most behavioral changes are observed at the dose of 0.03 mg/kg or higher (Ogura and Aigner, 1993; Buffalo et al., 1994; Harder et al., 1998). It is possible that higher doses might be more effective at disrupting PPI. Despite that, higher doses of MK-801 also induce a strong ataxia effect and parkinsonism, which could have disrupted subjects’ performance on PPI task (Crossman et al., 1989; Rupniak et al., 1992). Nevertheless, we observed, but not quantified, changes in monkey’s motor behavior typical of ataxia especially at 0.02 and 0.03 mg/kg. Motor disturbances, as ataxia, were previously reported in these doses in other monkey species (Boyce et al., 1991; Rupniak et al., 1992).

Prepulse inhibition was reduced after acute MK-801 administration, in agreement with previous studies with rodents (e.g., Feifel and Priebe, 1999; Long et al., 2006; Gururajan et al., 2011; Khella et al., 2014). This effect, however, is not observed after fourth test-weeks of testing, even with 2-week test interval, and monkeys’ PPI response becomes similar to the first trial. As seen in Figure 3B, there was no difference in startle response between both groups in the first test-week. On the other hand, prepulse+pulse startle responses were significantly different between groups in the first test-week, indicating a clear PPI disruption. Although some studies with PPI paradigm showed that MK-801 effects on rodents could be intensified after repeated sessions (Schulz et al., 2001; Gomes et al., 2014), our results indicate a reversal of PPI disruption in monkeys. Similar results were observed in rodents after successive injections of D_2_-like dopamine receptor agonists, such as amphetamine, cocaine, apomorphine, and quinpirole (Druhan et al., 1998; Byrnes and Hammer, 2000; Feifel et al., 2002; Culm and Hammer, 2004; Culm et al., 2004; Li et al., 2011), which was interpreted as a tolerance effect to these drugs. In the same way, tolerance was observed after repeated administration of MK-801 in rats’ motor activity (Dall’Olio et al., 1992). In addition, the repeated treatment with MK-801 decreased D_2_ receptor density (Dall’Olio et al., 1992). Since D_2_ agonist could lead to PPI disruption (Ralph and Caine, 2005), MK-801 tolerance in our study might be attributed to a decrease in D_2_ receptor density. However, effects of D_2_ agonists on PPI are species-dependent (Ralph and Caine, 2005).

Familiarization with test environment might also prevent MK-801-induced impairment on PPI. Several studies with learning and memory in rodents evidenced that NMDA antagonists lose their effects after the animal’s familiarization with the test environment (Shapiro and O’Connor, 1992; Uekita and Okaichi, 2005; Chan and McNally, 2009). Following this trend, Ennaceur et al. (2011) showed that MK-801 increased anxiety level in mice and this anxiogenic effect decreased after repeated exposures to the experimental apparatus. Other NMDA antagonists also induce similar effects when the animal is familiarized to the environment (Saucier et al., 1996; Roesler et al., 1998; Sanders and Fanselow, 2003). NMDA antagonists’ block hippocampal long-term potentiation (LTP) and this effect can be altered by pretraining or by familiarization to the test environment due to latent learning (Otnaess et al., 1999; Ennaceur et al., 2011). In addition, it is known that hippocampus is a structure related to PPI modulation (Swerdlow et al., 2001; Wolf et al., 2006; Kohl et al., 2013).

Although PPI reflects automatic preattentive process (Graham, 1975), higher level of cognitive process might be involved in PPI response. There is a positive correlation between increase PPI and superior strategy formation abilities in humans, which could be related to a more efficient early information process (Bitsios et al., 2006). Furthermore, several studies have demonstrated that directing attention to the prepulse signal increases PPI (see Li et al., 2009, for review). In our experimental design, subjects served as their own control, which leads to repeated trials. In this sense, familiarization with test conditions might explain changes in the effect of MK-801. It is also possible that PPI test familiarization may have increased attention to prepulse signaling, thus enhancing PPI response.

As reported by Li et al. (2011), PPI test consists of repeated presentations of mild stressors startle stimuli, and the constant repetition of this test may provoke alterations of drug effects on PPI response. Indeed, since MK-801 half-life is approximately 2 h (Hatfield et al., 1992), and our PPI sessions were always at least 2 weeks apart, the observed PPI reversal cannot be ascribed to the MK-801 effect alone. Therefore, the unexpected reversal of PPI disruption observed in the present study is suggestive of a drug-training interaction. Similar experiments with rats and mice found instead an increased MK-801 effect over repeated sessions (Schulz et al., 2001; Gomes et al., 2014). In that sense, our results underscore the neuropharmacological differences between rodents and primates (Maior et al., 2011) and the importance of non-human primates for basic research and preclinical testing.

## Conclusion

The present study demonstrated the effectiveness of MK-801 on PPI disruption in capuchin monkeys. In addition, our results suggest a drug-training interaction effect on the PPI response after repeated administration of MK-801. The habituation process observed in monkeys indicates that MK-801 adverse effects, such as sensorimotor gating impairments, may be reduced by MK-801 tolerance effect and after familiarization to PPI test. Therefore, future PPI tests with MK-801 should employ separate treatment groups to avoid habituation effects induced by extensive repeated sessions.

### Conflict of Interest Statement

The authors declare that the research was conducted in the absence of any commercial or financial relationships that could be construed as a potential conflict of interest.
